# The influence of timing of oocytes retrieval and embryo transfer on the IVF-ET outcomes in patients having bilateral salpingectomy due to bilateral hydrosalpinx

**DOI:** 10.3389/fsurg.2022.1076889

**Published:** 2023-01-05

**Authors:** He Yilei, Yang Shuo, Ma Caihong, Yang Yan, Song Xueling, Zhang Jiajia, Liu Ping, Li Rong, Qiao Jie

**Affiliations:** ^1^ Center for Reproductive Medicine, Department of Obstetrics and Gynecology, Peking University Third Hospital, Beijing, China; ^2^ National Clinical Research Center for Obstetrics and Gynecology, Peking University Third Hospital, Beijing, China; ^3^ Key Laboratory of Assisted Reproduction, Ministry of Education, Peking University, Beijing, China; ^4^ Beijing Key Laboratory of Reproductive Endocrinology and Assisted Reproductive Technology, Beijing, China

**Keywords:** salpingectomy, pregnancy outcome, laparoscopy, *in vitro* fertilization, embryo transfer

## Abstract

**Objective:**

The objective of the study was to investigate whether the sequence of oocyte retrieval and salpingectomy for hydrosalpinx affects pregnancy outcomes of *in vitro* fertilization and embryo transfer (IVF-ET) patients.

**Study Design:**

There were 1,610 bilateral hydrosalpinx patients who underwent laparoscopy salpingectomy and IVF-ET/intracytoplasmic sperm injection (ICSI) from January 2009 to December 2018. They were divided into two groups: oocyte retrieval first group: 235 accepted oocyte retrieval before salpingectomy; operation first group: 1,375 accepted oocyte retrieval after salpingectomy. The basic information and pregnancy outcomes of the two groups were compared. The pregnancy outcomes and influencing factors were analyzed among patients at different starting times of frozen-thawed embryo transfer (FET) or oocyte retrieval after the salpingectomy.

**Results:**

Patients in the oocyte retrieval first group had higher levels of basal follicle stimulating hormone and lower anti-Mullerian hormone levels (*P *< 0.05). There were no cases of pelvic infection or oocyte and embryo contamination after oocyte retrieval in the oocyte retrieval first group. In the frozen cycle, the clinical pregnancy and miscarriage rates of the oocyte retrieval first group were lower than those in the operation first group (*P *< 0.05), while the live birth rate was not significantly different (*P > *0.05). The live birth rates of patients ≥35 years old in the operation first group and the oocyte retrieval first group were not significantly different (29.3% vs. 23.3%, *P = *0.240). After adjusting for age and antral follicle count (AFC), oocyte retrieval 4–6 and 7–12 months after the operation had higher accumulated pregnancy rates [OR 1.439 (1.045–1.982), *P *= 0.026; OR 1.509 (1.055–2.158), *P *= 0.024] and higher accumulated live birth rates [OR 1.419 (1.018–1.977), *P *= 0.039; OR 1.544 (1.068–2.230), *P *= 0.021]. No significant difference was observed in the pregnancy outcomes of frozen embryo transfer at different times after salpingectomy (*P *> 0.05).

**Conclusion:**

No contamination of the embryo or infection was observed in patients who underwent oocyte retrieval before the operation. The interval between the operation and frozen embryo transfer did not affect the pregnancy outcomes. After adjusting for age and AFC, patients who underwent oocyte retrieval 4–6 and 7–12 months after the operation had higher accumulated pregnancy rates and live birth rates.

## Introduction

Tubal factor, as an indication of *in vitro* fertilization and embryo transfer, is the most common cause of female infertility, including hydrosalpinx, adhesions, and obstruction (1). Hydrosalpinx may cause inflammatory substances in the fluid to flow back to the uterine cavity and affect the implantation of fertilized embryos (2). Hydrosalpingeal fluid may also harm the microenvironment of the pelvis and adversely affect pregnancy outcomes. In a meta-analysis of 5,569 cycles (3), researchers found that the implantation and pregnancy rates of *in vitro* fertilization and embryo transfer (IVF-ET) in patients with hydrosalpinx were significantly lower, and the miscarriage rate was higher than in those without hydrosalpinx. However, performing salpingectomy before or after the oocyte retrieval is still controversial especially in patients of older age or with diminished ovarian reserve (DOR). Even in young women with normal ovarian reserve, some clinicians worry that salpingectomy may affect the ovarian reserve. If these patients choose controlled ovarian stimulation (COS) first, will the inflammatory factors of hydrosalpinx affect the quality of oocytes or embryos?

If retrieving the oocytes was chosen after laparoscopy and hysteroscopy, excluding tubal and uterine factors, some patients may encounter the challenge of salpingectomy affecting the ovarian blood flow and reducing the ovarian response during consequent IVF cycles (4). Some studies have shown (5) that salpingectomy does not impair the ovarian response during subsequent IVF treatment. Other patients chose to retrieve the oocytes first; however, hydrosalpinx may form an abscess or cause torsion (6), leading to infection and embryo contamination during controlled ovarian stimulation or oocyte retrieval (7). Few studies have illustrated when to start ovulation after laparoscopic salpingectomy has the least damage to the ovary and the best transplantation outcome in patients with hydrosalpinx.

This retrospective study analyzed the clinical characteristics and influencing factors of pregnancy outcomes of patients with bilateral hydrosalpinx who underwent laparoscopic surgery after oocyte retrieval and oocyte retrieval after the operation to explain the benefits and adverse effects of salpingectomy on IVF-ET pregnancy outcomes. We also compared the pregnancy outcomes of patients with different start times of ovulation induction and transplantation after surgery to help clinicians treat patients with hydrosalpinx. In addition, we also provided a theoretical basis for patients to make joint decisions to improve pregnancy outcomes.

## Materials and methods

### Subjects

We collected clinical data from patients with bilateral hydrosalpinx who underwent laparoscopic salpingectomy and received IVF or intracytoplasmic sperm injection (ICSI) in the reproductive center of the Peking University Third Hospital between January 2009 and December 2018.

Inclusion criteria: (1) Age ≤ 45 years old; (2) Hydrosalpinx was diagnosed by hysterosalpingography, B-ultrasound, or salpingectomy and confirmed by laparoscopic surgery; (3) Bilateral salpingectomy was performed in our center's reproductive surgery group.

Exclusion criteria: (1) Other factors that affect embryo implantation, such as uterine malformation, submucosal myoma, and intrauterine adhesions; (2) Patients with uncontrolled endocrine diseases, such as hyperprolactinemia and thyroid dysfunction; (3) Patients who had a history of pelvic surgery.

Tube factor is the most common infertility factor for IVF, and other factors, such as male factors, may also exist. Therefore, we did not exclude samples with other IVF/ICSI indications, such as male factors.

### Definition of groups

Based on the timing of laparoscopic surgery and the start of COS, patients were divided into two groups: (1) Oocyte retrieval first group: the time of oocyte retrieval was earlier than laparoscopic surgery, and frozen embryo transfer after the surgery; (2) Operation first group: the operation time was earlier than oocyte retrieval.

Clinical pregnancy was defined as the presence of a gestational sac, with or without a fetal heartbeat as examined by transvaginal ultrasound examination 4 weeks after embryo transfer. Live birth was defined as delivery of any viable neonate who was 28 weeks of gestation or older. The accumulated clinical pregnancy/live birth rate is the ratio of patients who had at least one presence of a gestational sac/live birth baby after the transfer of all fresh and frozen-thawed/warmed embryos to the ovarian stimulation patients in each group.

The study was approved by the ethics committee of the reproductive center of the Third Hospital of Peking University, No. 2008013.

### Sample collection

On the second day of menstruation/withdrawal bleeding and the day of human chorionic gonadotropin (hCG) injection, the patient's serum was tested for the levels of follicle stimulating hormone (FSH), luteinizing hormone (LH), estradiol (E_2_), progesterone (*P*) and anti-Mullerian hormone (AMH) by Immulite 2000 (Los Angeles, California, United States). Quality control was provided by Bio-RAD Laboratory (Lyphochek Immunoassay Plus Control, Trilevel), Catalog No. 370, Batch No. 40300. The inter-and intra-assay coefficients of variation were less than 10% and 15%, respectively. We compare FSH and AMH based on the criterion of decreased ovarian reserve. Because the results may fluctuate due to the limitation of the detection method, the means of FSH and AMH may have limited clinical significance.

### Laparoscopic bilateral salpingectomy and IVF/ICSI-Et

Laparoscopic bilateral salpingectomy was performed according to the routine operation of the reproductive center of the Third Hospital of Peking University.

IVF/ICSI-ET process: on the second day of menstruation/withdrawal bleeding, serum hormone examination showed FSH < 12 IU/L and E_2 _< 200 pmol/L; hCG was negative; B-ultrasound showed there were no large follicles (diameter ≥10 mm) or cysts in bilateral ovaries and the thickness of endometrium was ≤7 mm; gonadotropins (Gn) were activated, including recombinant FSH (recombinant human follicle stimulating hormone for injection, Merck Serono, Switzerland; recombinant follicle stimulating hormone *β* injection, Organon, Netherlands), urogenic FSH (urinary follicle stimulating hormone for injection, Institut biochimique SA, Switzerland), and urogenic human menopausal gonadotropin (hMG) (Lizhu Pharmaceutical Co., Zhuhai). After 4–5 days of Gn application, vaginal B-ultrasound was used to monitor the growth of follicles, and the levels of serum LH, E_2_, and *P* were checked. When three follicles ≥ 18 mm (follicles ≥ 17 mm were used for the antagonist regimen) were detected, human chorionic gonadotropin (r-hCG, Adze, 250 μg) was injected that night and then the oocytes were retrieved under the guidance of transvaginal ultrasound 36–38 h later. All patients were given luteal support after oocyte retrieval and progesterone gel was used in the vagina (9% vaginal progesterone gel, Swiss Merck Serono). One or two embryos were transferred 3/5 days after oocyte retrieval and the remaining embryos were cryopreserved.

The endometrial preparation in the frozen cycle included the natural cycle or artificial cycle, which was carried out according to the diagnosis and treatment routine of our center. In the natural cycle/ovulation induction cycle, 20–40 mg/day of didroxyprogesterone was given from the day of transplantation. The artificial cycle was started on 1–3 days of menstruation, with 6 mg/day of oral progesterone. The endometrial condition was monitored by ultrasound after taking medicine for 10 days. Progesterone conversion (progesterone 40 mg/day, vaginal progesterone gel 90 mg/day) was performed after the endometrial thickness of 8 mm, which was set as D0. The embryo is transferred on D3/D5. The transfer rate was calculated as the number of transferred cycles divided by the total number of cycles. The miscarriage rate was defined as the number of miscarriages per woman. Live birth was defined as the delivery of a viable fetus at >28 weeks of gestation. Clinical pregnancy was defined as the accumulated number of clinical pregnancies per woman randomized (demonstrated by serum hCG positive twice and confirmed by ultrasonographic visualization of one or more gestational sacs).

### Statistical analysis

SPSS 26.0 software was used for data analysis and statistical processing. The measurement data were expressed as the mean ± SD and the data were tested for normality and homogeneity of variance. T test was used to compare the mean between the normal distribution and homogeneity of variance groups; otherwise, the nonparametric Wilcoxon rank sum test was used. Chi-square and Fisher exact tests were applied to evaluate the categorical variables between different groups. All tests were two-sided, and *P* < 0.05 was considered a statistically significant difference.

## Result

### The characteristics of the study groups

**Figure 1:**
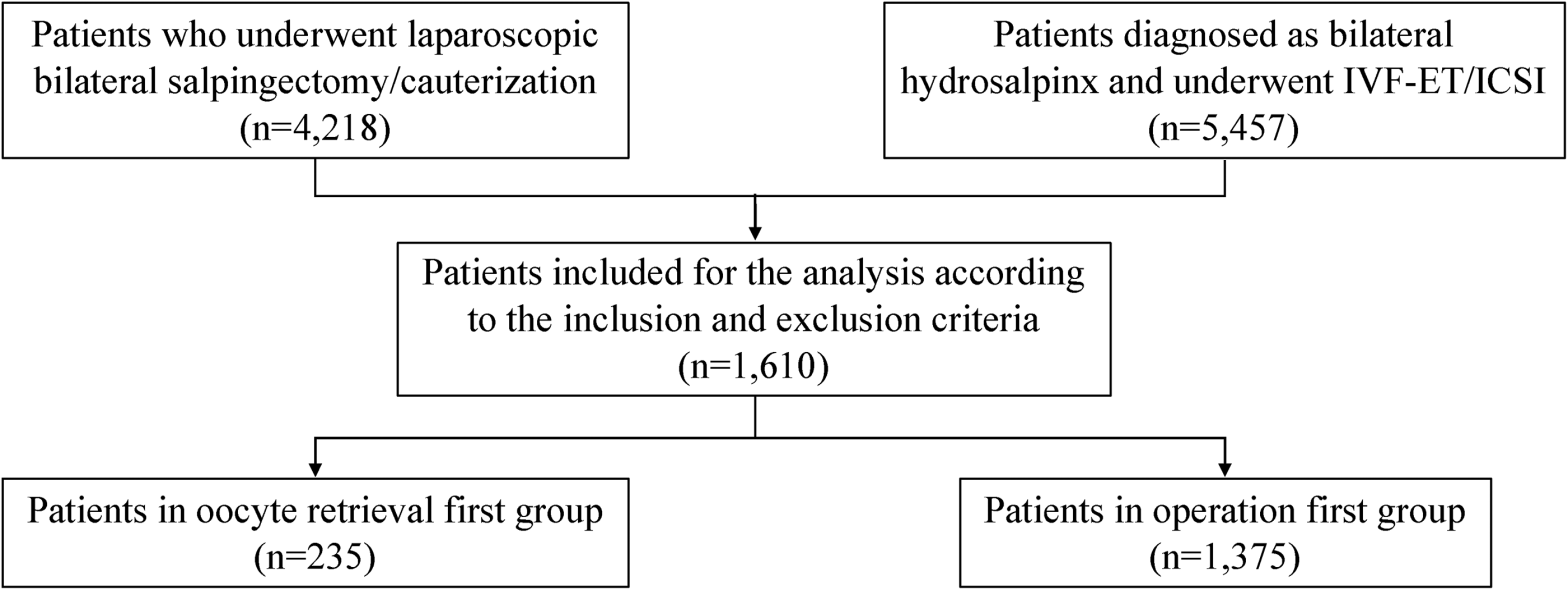
Flowchart illustrating the inclusion of cases in the study.

The clinical data of 4,218 patients who underwent laparoscopic bilateral salpingectomy due to bilateral hydrosalpinx were collected. A total of 5,457 patients with bilateral hydrosalpinx were diagnosed and treated with IVF-ET/ICSI. According to the inclusion and exclusion criteria, a total of 1,610 patients were included in this study, with 235 cases in the oocyte retrieval first group and 1,375 cases in the operation first group (Figure 1).

The general characteristics of the two groups were listed in Table 1. Compared with the operation first group, the oocyte retrieval first group had higher levels of basal FSH and lower AMH levels (*P *< 0.05). The protocol of controlled ovarian hyperstimulation was different and the number of Gn administration days was shorter in the oocyte retrieval first group (*P *< 0.05). There were no cases of pelvic infection or oocyte and embryo contamination after oocyte retrieval in the oocyte retrieval first group.

**Table 1:** The general characteristics of two groups.

	Oocyte retrieval first group (*n* = 235) mean ± SD	Operation first group (*n* = 1,375) mean ± SD	*P* value
Age (years)	33.4 ± 5.3	33.4 ± 4.5	0.992
Infertility years (years)	5.3 ± 4.3	5.8 ± 4.0	0.077
Primary Infertility	53.6% (126/235)	46.0% (633/1,375)	0.005
BMI (kg/m^2^)	22.3 ± 3.2	22.7 ± 3.4	0.075
FSH > 9 mIU/ml	23.0% (54/235)	14.8% (203/1,375)	0.004
AMH > 1.1 ng/ml	37.9% (89/235)	84.7% (1,165/1,375)	<0.001
AFC (*n*)	9.8 ± 6.4	9.8 ± 4.8	0.984
Protocols of controlled ovarian stimulation			
Ultralong GnRH agonist	9.8% (23/235)	12.8% (176/1,375)	
Long GnRH agonist	28.1% (66/235)	42.5% (585/1,375)	
Short GnRH agonist	12.3% (29/235)	9.7% (134/1,375)	
GnRH antagonist	49.8% (117/235)	34.9% (480/1,375)	
Gn days (*d*)	10.9 ± 2.6	11.4 ± 2.4	0.005
Gn dose (U)	2,956.0 ± 1,309.8	3,057.3 ± 1,320.7	0.277
No. of oocytes (*n*)	12.1 ± 9.1	12.1 ± 7.0	0.940
ICSI rate	19.9% (46/231)	22.1% (301/1,365)	0.254
MII rate	13.4% (380/2,844)	15.7% (2605/16,587)	0.594

The patients were divided into different groups according to the basic FSH ≤ 9 mIU/ml and serum AMH ≤ 1.1 ng/ml.

BMI, body mass index = weight (kg)/height (m)^2^; FSH, follicle stimulating hormone; AMH, anti-Mullerian hormone; AFC, antral follicle count; GnRH, gonadotropin-releasing hormone; Gn, gonadotropins; IVF, in vitro fertilization; ICSI, intracytoplasmic sperm injection; Half, half IVF and half ICSI.

The pregnancy outcomes of first-time embryo transfer among the oocyte retrieval first group and the operation first group were listed in Table 2. There was no difference in the clinical pregnancy rate or live birth rate between the two groups after the first embryo transfer. The miscarriage rate was higher in the operation first group (6.8% vs. 1.0%, *P *= 0.001).

**Table 2:** Pregnancy outcomes of first-time embryo transfer among oocyte retrieval first group and operation first group.

Outcomes	Oocyte retrieval first group (*n* = 235)	Operation first group (*n* = 1,375)	*P* value
Clinical pregnancy rate	38.7% (77/199)^a^	43.3% (563/1,301)^a^	0.224
Live birth rate	37.7% (75/199)	36.4% (474/1,301)	0.717
Miscarriage rate	1.0% (2/199)	6.8% (89/1,301)	0.001

^a^ There were 23 patients do not have any available embryo, and 13 patients canceled frozen embryo transfer in the oocyte retrieval first group, 70 patients do not have any available transfer embryo, and 4 patients canceled frozen emryo transfer in the operation first group.

### Outcomes of patients with bilateral hydrosalpinx in different age groups

**Figure 2:**
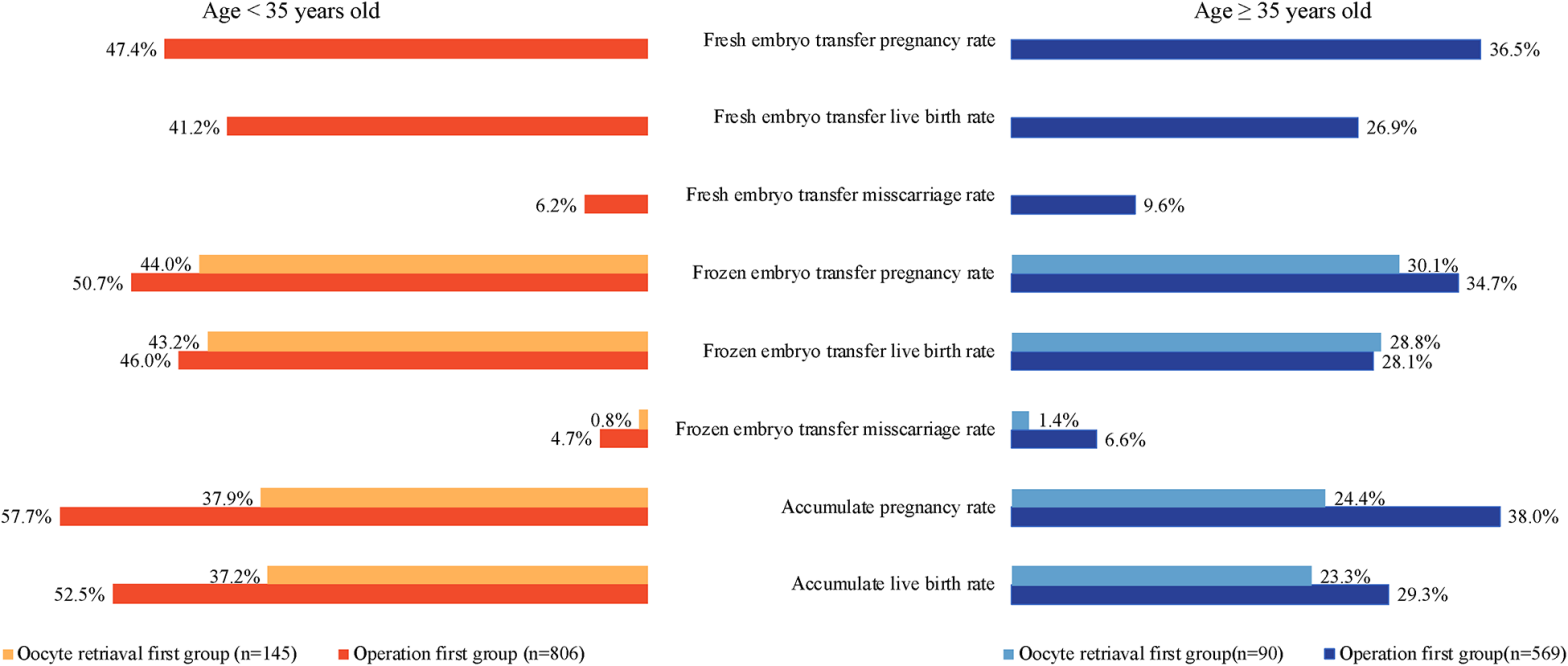
Outcomes of patients with bilateral hydrosalpinx in different age groups.

In Figure 2, the patients were divided into two groups according to age (8). Patients less than 35 years old had higher clinical pregnancy rates, live birth rates, and lower miscarriage rates in the fresh and frozen cycles than patients ≥35 years old in both the oocyte retrieval first group and the operation first group (*P < *0.05). Patients <35 years old in the operation first group had higher accumulated clinical pregnancy rates (57.7% vs. 37.9%, *P < *0.001) and live birth rates (52.5% vs. 37.2%, *P = *0.001) than patients in the oocyte retrieval first group. Patients ≥35 years old in the operation first group had higher accumulated clinical pregnancy rates than patients in the oocyte retrieval first group (38.0% vs. 24.4%, *P = *0.013); however, the live birth rates were not significantly different (29.3% vs. 23.3%, *P = *0.240).

### Interval between starting IVF cycles, frozen embryo transfer, and the time of laparoscopic surgery

After laparoscopic surgery, IVF cycles were started at different times of ≤3 months, 4–6 months, 7–12 months, and more than a year. The longer the time between operation and IVF and the older the patient, the longer the infertility years and the lower the ovarian reserve (*P < *0.05, Table 3). These patients had shorter days of Gn induction, a larger dosage of Gn, fewer oocytes retrieved, and lower accumulated pregnancy and live birth rates (*P < *0.05).

**Table 3:** Oocyte retrieval initiated at different time after laparoscopic surgery.

	≤3 months (*n* = 560)	4-6 months (*n* = 296)	7–12 months (*n* = 228)	>1 year (*n* = 291)	*P* value
	Mean ± SD	Mean ± SD	Mean ± SD	Mean ± SD	
Age (years)	32.1 ± 4.4	32.7 ± 4.3	34.4 ± 4.3	35.8 ± 4.1	<0.001
Infertility years (years)	5.3 ± 3.8	5.6 ± 4.1	6.4 ± 4.0	6.5 ± 4.1	<0.001
BMI (kg/m^2^)	22.7 ± 3.2	22.5 ± 3.4	22.7 ± 3.4	23.0 ± 3.6	0.440
FSH > 9 mIU/ml	12.1% (68/560)	11.5% (34/296)	20.6% (47/228)	18.6% (54/291)	0.002
AMH > 1.1 ng/ml	87.3% (489/560)	89.9% (266/296)	61.4% (140/228)	80.1% (233/291)	0.024
AFC (*n*)	10.1 ± 4.7	10.1 ± 4.2	9.5 ± 4.8	9.3 ± 5.4	0.098
Gn days (d)	11.7 ± 2.5	11.5 ± 2.4	11.1 ± 2.4	11.0 ± 2.3	<0.001
Gn dose (U)	2,941.7 ± 1,279.6	2,943.8 ± 1,238.6	3,268.8 ± 1,457.6	3,228.0 ± 1,334.0	0.001
Number of oocytes (n)	12.9 ± 6.9	12.7 ± 7.2	11.2 ± 6.0	10.5 ± 7.4	<0.001
Fresh-embryo transfer rate	84.1% (471/560)	85.8% (254/296)	84.6% (193/228)	73.2% (213/291)	<0.001
Fresh-embryo clinical pregnancy rate	42.7% (201/471)	50.8% (129/254)	39.4% (76/193)	38.0% (81/213)	0.023
Fresh-embryo live birth rate	35.0% (165/471)	42.5% (108/254)	33.7% (65/193)	30.0% (64/213)	0.036
Fresh-embryo miscarriage rate	7.6% (36/471)	8.3% (21/254)	5.7% (11/193)	8.0% (17/213)	0.754
Accumulated clinical pregnancy rate	55.2% (309/560)	56.4% (167/296)	41.2% (94/228)	38.2% (111/291)	<0.001
Accumulated live birth rate	48.4% (271/560)	49.7% (147/296)	36.4% (83/228)	30.7% (89/291)	<0.001

BMI, body mass index = weight (kg)/height (m)^2^; FSH, follicle stimulating hormone; AMH, anti-Mullerian hormone; AFC, antral follicle count; Gn, gonadotropins; IVF, in vitro fertilization; ICSI, intracytoplasmic sperm injection; Half, half IVF and half ICSI.

After adjusting for age and AFC, significant differences in pregnancy outcomes between IVF cycles started at different times after the operation were observed (Table 4). Compared with the oocyte retrieval ≤3 months group, oocyte retrieval 4–6 and 7–12 months after the operation had higher accumulated pregnancy rates [OR 1.439 (1.045–1.982), *P *= 0.026; OR 1.509 (1.055–2.158), *P *= 0.024] and higher accumulated live birth rates [OR 1.419 (1.018–1.977), *P *= 0.039; OR 1.544 (1.068–2.230), *P *= 0.021].

**Table 4:** Logistic regression analysis of factors associated with pregnancy outcomes.

	Accumulated clinical pregnancy		Accumulated live birth	
	OR (95%CI)	*P* value	OR (95%CI)	*P* value
Age	0.914 (0.889–0.939)	<0.001	0.895 (0.870–0.921)	<0.001
AFC	1.043 (1.018–1.068)	0.001	1.036 (1.012–1.062)	0.004
Oocyte retrieval ≤3 months after the operation	Reference	0.016	Reference	0.043
Oocyte retrieval 4–6 months after the operation	1.439 (1.045–1.982)	0.026	1.419 (1.018–1.977)	0.039
Oocyte retrieval 7–12 months after the operation	1.509 (1.055–2.158)	0.024	1.544 (1.068–2.230)	0.021
Oocyte retrieval >12 months after the operation	0.965 (0.659–1.414)	0.855	1.050 (0.705–1.562)	0.811

AFC, antral follicle count.

Patients in the oocyte retrieval first group were divided into two groups according to frozen embryo transfer ≤3 or >3 months after salpingectomy. The general characteristics of these two groups and pregnancy outcomes were not significantly different (*P *> 0.05). (Table 5).

**Table 5:** Thawing and transplantation at different time after laparoscopic surgery.

	≤3 months (*n* = 146)	>3 months (*n* = 52)	*P* value
	Mean ± SD	Mean ± SD	
Frozen embryo clinical pregnancy rate	39.0% (57/146)	38.5% (20/52)	0.941
Frozen embryo live birth rate	37.7% (55/146)	38.5% (20/52)	0.920
Frozen embryo miscarriage rate	1.4% (2/146)	0% (0/52)	0.396

## Discussion

Over the last 30 years, with the continuous improvement of IVF-ET success rates, the potentially harmful effects of hydrosalpinx on pregnancy outcomes have become evident (9). Salpingectomy helps optimize the procedure of oocyte retrieval procedure and reduce the risk of infection (10). The sequence of salpingectomy and oocyte retrieval is a common problem in clinical practice and lacks study. Whether the start time of promoting ovulation after salpingectomy affects the outcome of transplantation remains an interesting topic for clinical workers and patients. In this study, we analyzed the clinical characteristics and pregnancy outcomes of patients with bilateral hydrosalpinx who underwent oocyte retrieval before or after laparoscopic surgery. To our knowledge, this study is the first to compare the pregnancy outcomes of patients with different start times of oocyte retrieval or embryo transfer after the surgery. This was done to provide a theoretical basis for patients with hydrosalpinx to improve the pregnancy outcome of IVF-ET, particularly for long-term fertility outcomes such as accumulated live birth rates.

### The sequence of salpingectomy and COS

Previous studies have shown that hydrosalpinx fluid flow may affect embryo implantation through mechanical cleaning of the uterine cavity (11). The liquid contains microorganisms, toxic substances, lymphocytes, cytokines, prostaglandins, etc., which may damage the ovaries and indirectly affect the quality of oocytes and the pelvic microenvironment and directly return to the uterine cavity, affecting embryo or endometrium and embryo implantation (12). The presence of hydrosalpinx increases the risk of pregnancy loss and treatment can reduce this risk (2). Salpingectomy, salpingostomy, and tubal occlusion were possible management options for reproductive women with hydrosalpinx. However, low clinical pregnancy and live birth rates have been reported among patients who underwent proximal tubal occlusion, and ectopic pregnancy rates were reported among patients who underwent salpingostomy, or distal tubal plastic surgery (13). Laparoscopic salpingectomy is recommended for patients with hydrosalpinx to effectively treat tubal infertility (14). Previous studies have illustrated that salpingectomy before assisted reproductive technology probably increases the clinical pregnancy rate compared to no surgery in women with hydrosalpinges (15). Cochrane's review emphasizes that patients with tubal infertility should undergo laparoscopic salpingectomy (or at least tubal obstruction) before IVF-ET to obtain a high pregnancy rate (16). One meta-analysis showed that salpingectomy does not impair the ovarian response during subsequent IVF treatment (4). Another meta-analysis on the effect of bilateral salpingectomy/cauterization on ovarian reserve suggested that there were no unified conclusions from different studies to date (17). Laparoscopy and oocyte retrieval at our center were performed by experienced doctors with unified rules, which is the strength of our study. The laboratory also has unified and strict professional management rules. No postoperative infection, pelvic inflammatory disease, or inability to implant due to oocyte and embryo contamination occurred in the patients. Our retrospective study showed that the clinical pregnancy rate and live birth rate of first embryo transfer were not significantly different between the oocyte retrieval first and operation first groups. Therefore, we concluded that the success rate of IVF after surgery performed by a standardized trained reproductive gynecologist was satisfactory. In addition, IVF before surgery was safe and did not increase the risk of pelvic infection or embryo contamination.

### Pregnancy outcomes in different age groups patients

Some patients may encounter challenges with few available embryos in the frozen embryo transfer cycle after the surgery and need COS, in addition to an older age. Chen et al. conducted a retrospective study among IVF-ET patients aged 35–39 years, which showed that salpingectomy might decrease the antral follicle count but not the live birth rate (18). Concerning the influence of age on IVF outcomes, our study divided patients into two groups, <35 and ≥35 years. Our study showed that the accumulated pregnancy rates and live birth rates were higher in the operation first group among patients <35 years old; however, the difference in the accumulated live birth rates was not significant among patients ≥35 years. A previous study pointed out that the pregnancy loss rate in women of 10–14 years was 3.9%, increasing gradually with age to 26.9% in pregnant women of 45–49 years, a 6.9-fold increase (19). Since the rate of pregnancy loss due to the poor quality of the embryo was higher in women of older age and the surgery would delay the precious time for oocyte retrieval, freezing embryos before surgery is also a good option. Therefore, in patients ≥35 years old, with decreased oocyte number and quality, expected pelvic adhesions, and concerns about surgical damage to ovarian function, we can choose to cryopreserve the embryo first and then perform laparoscopic surgery before frozen embryo transfer to obtain satisfactory pregnancy outcomes.

### Timing of COS in patients with bilateral hydrosalpinx after laparoscopic surgery

In guiding the timely pregnancy of these patients, the patient's infertility years and the degree of tubal lesions should be fully considered to individualize the interval of ovulation induction and operation to improve the pregnancy rate (20). In this study, we found that patients who started COS at a longer interval after surgery had an older age, longer infertility years, and lower ovarian reserve. The number of oocytes retrieved was lower among these patients. The pregnancy outcomes were not satisfactory in patients who started COS longer after the operation. After age and AFC were adjusted, the results showed that compared with patients who started IVF ≤3 months after the operation, patients in the 4–6 and 7–12 months groups had better pregnancy outcomes. In accordance with previous studies, a lower level of the ovarian reserve marker AMH was observed a few months after salpingectomy in patients with IVF-ET than in the control group and rose again (21, 22). However, other studies showed that the level of AMH in patients who underwent salpingectomy was not significantly different from that in patients without salpingectomy (23, 24). Presently, the conclusion was not unified because the intervals of AMH measurement and salpingectomy were different (25), and different studies included patients who underwent salpingectomy for different purposes. The strength of our study is that the purpose of salpingectomy was all for hydrosalpinx, and the mesosalpinx was maximally preserved at the same level. The duration and energy of electrocoagulation were carefully used to achieve coagulation simultaneously, as salpingectomy might damage the vascular perfusion of the ovary (26).

### Timing of FET after laparoscopic surgery in patients retrieving oocytes first

Some researchers have suggested that the first-line treatment for young women less than 35 years old with minor tubal pathology is tubal surgery, and IVF should be offered if the patient is >38 years old, if moderate to severe tubal disease is present, and if it has been more than 12 months postsurgery (27). Our research suggested that for those who had concerns about the damage to the ovarian reserve caused by the operation, cryopreservation before the operation and frozen embryo transfer can be considered. Comparing the characteristics and outcomes of patients who received frozen embryo transfer at different times after the operation, the number of oocytes retrieved was lower in patients with an interval of more than 3 months, but there were no significant differences in pregnancy outcomes. The results suggested that in our clinical work, the start-up time of the frozen cycle after the operation did not affect the pregnancy outcomes. The drawbacks of this study included the number of patients who underwent frozen embryo transfer was only a few. Thus, further research could enlarge the number of patients and make a solid conclusion.

## Conclusion

In summary, the live birth rate of first-time embryo transfer between the operation first group and oocyte retrieval group was not significantly different. Patients less than 35 years old in the operation first group had higher accumulated live birth rates than patients in the oocyte retrieval first group; however, this difference was not significant in patients ≥35 years old. The longer the time between operation and IVF, the fewer the number of oocytes retrieved and the lower accumulated pregnancy and live birth rates. After adjusting for age and AFC, patients who underwent oocyte retrieval 4–6 months and 7–12 months after the operation had higher accumulated pregnancy rates and live birth rates. No contamination of the embryo or infection was observed in patients who underwent oocyte retrieval before the operation. The interval between frozen embryo transfer and the operation did not affect the pregnancy outcomes. This study provides a reference for the treatment of hydrosalpinx patients before salpingectomy and a theoretical basis for helping patients make joint decisions to obtain better pregnancy outcomes.

## Data Availability

The raw data supporting the conclusions of this article will be made available by the authors, without undue reservation.
